# Does Modulation of an Epigenetic Clock Define a Geroprotector?

**DOI:** 10.20900/agmr20220002

**Published:** 2022-03-29

**Authors:** Nicholas J. Schork, Brett Beaulieu-Jones, Winnie Liang, Susan Smalley, Laura H. Goetz

**Affiliations:** 1 Department of Quantitative Medicine, The Translational Genomics Research Institute (TGen), 445 North Fifth Street, Phoenix, AZ 85004, USA; 2 Net.bio Inc, Los Angeles, CA 90403, USA; 3 Department of Biomedical Informatics, Harvard University, Cambridge, MA 02115, USA; 4 Department of Psychiatry and Biobehavioral Sciences, The University of California Los Angeles, Los Angeles, CA 90095, USA

**Keywords:** geroprotectors, the geroscience hypothesis, clinical trials, epigenetic clocks, biomarkers

## Abstract

There is growing interest in the development of interventions (e.g., drugs, diets, dietary supplements, behavioral therapies, etc.) that can enhance health during the aging process, prevent or delay multiple age-related diseases, and ultimately extend lifespan. However, proving that such ‘geroprotectors’ do what they are hypothesized to do in relevant clinical trials is not trivial. We briefly discuss some of the more salient issues surrounding the design and interpretation of clinical trials of geroprotectors, including, importantly, how one defines a geroprotector. We also discuss whether emerging surrogate endpoints, such as epigenetic clocks, should be treated as primary or secondary endpoints in such trials. Simply put, geroprotectors should provide overt health and disease prevention benefits but the time-dependent relationships between epigenetic clocks and health-related phenomena are complex and in need of further scrutiny. Therefore, studies that enable understanding of the relationships between epigenetic clocks and disease processes while simultaneously testing the efficacy of a candidate geroprotector are crucial to move the field forward.

## INTRODUCTION

The development of drugs, diets, activities, etc. that sustain health throughout the aging process, increase vitality and ultimately enhance longevity has been on the minds of humans for centuries [1–3]. Not only is this interest rooted in an innate individual desire to live a long and healthy life, but, more generally, there is a growing consensus among biomedical scientists that by identifying interventions that modulate some basic mechanisms of aging, a number of age-related diseases can be prevented, or at least have their onset slowed. Interventions that do indeed slow the aging rate have the potential to prevent or mitigate damage to the body, which, accumulated over time, creates vulnerability to disease, thereby creating unprecedented opportunities for achieving healthcare efficiency. A number of approaches to the development of such ‘geroprotectors’ have been proposed, including those that seek to mimic the beneficial molecular effects of caloric restriction [4], ‘senolytic’ approaches which attempt to clear out senescent cells and the deleterious age-related debris that they secrete [5,6], reprogramming approaches exploiting insights into stem cell biology and cellular rejuvenation [7–13], and approaches based on the identification of circulating factors associated with healthy youth that can be literally infused into older individuals [12,14]. However, despite this interest and the growing number of emerging approaches to the development of geroprotectors, testing and proving their value are complicated and raise a number of important questions.

In this brief review we describe some issues of fundamental importance to the development and testing of geroprotectors. We raise a number of questions that, if addressed, could help set a framework within which geroprotectors can be evaluated. Primary among these questions are concerns about the use of surrogate measures such as epigenetic clocks as primary endpoints in relevant clinical trials. We also consider the mechanistic links between epigenetic clocks (and other biological clocks) and disease processes which, by definition, a geroprotector should mitigate. These mechanistic links bear on, for example, the length of time one would need to be on a geroprotector before its beneficial effects take hold and whether the beneficial processes that come with the positive modulation of an epigenetic clock are independent of processes associated with widely accepted clinical and subclinical measures of disease, such as cholesterol, memory loss and obesity level. Ultimately, these questions and considerations should motivate greater discussion about the design of appropriate clinical trials for geroprotectors.

## THE GEROSCIENCE HYPOTHESIS

The development of geroprotectors is rooted in the ‘geroscience hypothesis’ which posits that interventions that target and ultimately modulate or slow down very basic mechanisms of aging could reduce susceptibility to many age-related diseases simultaneously [1–3,15,16]. Such a hypothesis is consistent with the belief that the set of genes contributing to the aging process may be different from the set of genes contributing to any one age-related disease, since some aspects of an age-related disease are a consequence of aging itself. As such, genes implicated in aging have broad effects, rather than being disease specific [17,18]. Blockbuster drugs such as atorvastatin or lisinopril, which were designed specifically to reduce cholesterol and blood pressure level, respectively, and thereby only prevent heart disease and hypertension without having broader effects on multiple age-related conditions, are not by definition geroprotectors. A number of very compelling reports have been published that expose and characterize basic mechanisms or hallmarks of aging that, if amenable to, e.g., pharmacological modulation, could lead to the development of geroprotectors [2,3,19]. In addition, as noted, many candidate geroprotectors have been proposed that actually appear to modulate some of these hallmarks [4–14,20–22]. Despite this, there is consensus that more sophisticated studies are needed in order to truly test the geroscience hypothesis for any given candidate geroprotector [2,3,19]. The reasons for this are somewhat obvious in that appropriate studies would have to focus on the impact that a candidate geroprotector has on multiple age-related diseases and not just one, per the definition of a geroprotector. This can be complicated and take a considerable amount of time. For example, tracking individuals receiving a geroprotector and those receiving a comparator intervention or placebo over a long enough period of time to show that rates of different diseases are reduced among individuals receiving the geroprotector could take years [23].

The consideration of multiple disease endpoints in the evaluation of a geroprotector is not unprecedented however, as it is essentially the strategy to be exploited in the expensive and lengthy, ‘Targeting Aging with Metformin’ (TAME) trial focusing on metformin as a candidate geroprotector [24,25]. As an alternative to the use of multiple disease incidence measures that may take a long time to gather appropriately, it has been argued that the use of biomarkers that capture various aging hallmarks, as well as general measures of the aging rate, could be used in relevant trials. Thus, these measures, if shown to be modulated by an intervention, could provide evidence that something fundamental and relevant to the aging process is affected by that intervention. Proof that an intervention modulated these measures would at the very least qualify that intervention as a candidate geroprotector that could be evaluated in longer-term disease incidence-based trials [3,16,21,22,26,27]. The current pool of relevant biomarkers, which includes transcript [28,29] and protein profiling [30,31] as well as telomere length measures [32–34], are being complemented by various DNA methylation-based (or epigenetic) clocks designed to specifically measure the aging rate [35–41]. However, epigenetic clocks and related measures of the aging rate need a great deal more scrutiny before they should be considered as a primary endpoint in at least early-stage clinical trials of candidate geroprotectors.

## BIOMARKERS AND AGING CLOCKS

DNA methylation-based or epigenetic clocks consider measuring individual aging rates by more or less counting changes in CpG sites (gains or losses in methylation) that occur as one ages [38]. A number of epigenetic clocks have been proposed, with the differences between them reflecting the use of different numbers and configurations of methylation target (CpG) sites in the genome, different cell types, and different methods/data for training and ultimately scoring them from a statistical analysis perspective (e.g., how chronological age is factored in to the measure) [38,40–44]. Importantly, as discussed in detail below, the differences in the way epigenetic clocks have been constructed have led to differences in the strength of the correlations between them, as well as with independent measures of aging and health and disease.

As noted, it has been suggested that potential geroprotectors could be tested to see if they reverse or slow an epigenetic clock and hence the aging rate in a clinical trial and thus save the trial from having to collect complicated health measures and disease onset outcomes [15,16,23]. In fact, a few very recent trials of potential geroprotectors have found evidence for positive changes in specific epigenetic clocks, suggesting there is potential for this approach [45–48]. We note that there is considerable research exploring epigenetic clocks in non-human species that also makes the case for their use in studies of the effects of geroprotectors [41], but we confine our attention to studies of humans.

Despite their potential, there are at least four issues plaguing the use of currently available epigenetic clocks as primary endpoints in short term trials of geroprotectors. First, the many available epigenetic clocks are only weakly to moderately correlated [42,43,49–54], suggesting that either they measure different aspects of the aging rate, or there is something even more fundamental than what they are capturing that could tie them together and ultimately better reflect the aging rate. In this light, one recent study did find evidence for a common set of molecular physiologic phenomena, based on gene expression patterns, that may be common immediate consequences or causes of many epigenetic clocks, although a great deal of variation among the clocks was still observed [49]. In addition, a few recent studies suggest that combining available epigenetic clocks may lead to more sensitive measures of the aging rate. However, these aggregated clocks, especially those that consider multiple tissues, have yet to be evaluated in independent studies and may be hard to evaluate given problems with tissue accessibility in living humans [43,49]. In the context of clinical trials of geroprotectors, it could be asked that if different epigenetic clocks truly capture different facets of the molecular physiologic determinants of aging and aspects of health as a result, then by definition should a geroprotector modulate all or at least many of them?

Second, many of the available epigenetic clocks have been shown to be predictive of mortality and morbidity in both case-control and retrospective longitudinal cohort studies. However, they do not necessarily outperform other measures of the aging rate in appreciable ways, such as telomere length, frailty assessments, functional indices, and clinical chemistry composites, and are only moderately correlated with these measures [44,50–56]. In addition, epigenetic clocks do not correlate well with other traditional clinical and subclinical measures of health [42,44], although at least one epigenetic clock has been designed to capture variation associated with different subclinical measures of health: the ‘DunedinPoAm’ (‘Dunedin Pace Of Aging Methylation’) measure [57]. This raises the question of whether or not one should put stock in a geroproector that essentially modulates an aging clock but does not actually impact any of the numerous clinical and subclinical measures that are currently associated with health and health trajectory (e.g., blood pressure, lipid and glucose levels, cardiac, kidney and lung function, sleep quantity and quality, etc.). In addition, a study of a geroprotector could indicate that its use is indeed associated with positive changes in an epigenetic clock, but only with a small subset of a more comprehensive set of health measures. This would then suggest that either: (1) the epigenetic clock(s) used only captures components of the aging process as discussed previously; (2) the chosen health measures are not good indicators of general health and are therefore peripheral in some way to what is essential in preserving health in the long term; (3) the epigenetic clock(s) reflect or tap into health processes that are somehow more fundamental to longevity in a way that does not discount the value of traditional health measures but somehow renders the signs and symptoms associated with those traditional health measures (e.g., elevated cholesterol or high blood pressure) benign; or (4) The candidate geroprotector is in fact not a geroprotector since it does not positively influence multiple age-related disease processes.

Third, it is likely that epigenetic clocks are the consequences of other age-related health processes and not contributors or the causes of those processes. Thus, the *causal* relationships between mechanistic phenomena determining epigenetic clocks and health-preserving processes in general must be put into perspective, especially if those epigenetic clocks are to be used as primary endpoints in clinical trials of candidate geroprotectors [15,16,38,40]. It should be emphasized that if changes to health processes are accompanied by changes in an epigenetic clock, then important questions arise as to how long an interval is likely to occur between changes in health processes and those changes reflected in an epigenetic clock, as well as how pronounced those changes have to be before they are reflected in a clock. Most studies linking changes in epigenetic clocks with health measures have involved longitudinal cohort studies with infrequent, often inconsistent, yet lengthy, time intervals between them [38,40,43,44,49,56–59]. In addition, only a few small and probably statistically underpowered clinical trials have resulted in evidence of trends indicating that changes in health parameters accompany changes in an epigenetic clock [45–47].

Fourth, the length of time a geroprotector needs to be administered in order for it to induce positive changes in health is of crucial importance for putting into perspective the use of epigenetic clocks as primary endpoints in clinical trials. Thus, one could ask if slowing of the aging rate as indicated by an epigenetic clock does not accompany immediate health changes (e.g., blood pressure lowering), then how does it bypass the need for these health changes in positively impacting longevity and how long does one need to be on a geroprotector before it reduces age-related disease susceptibility or severity? That is, can the degree of slowing or change in an epigenetic clock associated with geroprotector use anticipate long term health benefits? How long might it take for the geroprotector to essentially ‘remodel’ or positively impact an individual’s molecular and organismal-level physiology in a way that will sustain (better) health going forward? What can the changes in epigenetic clocks say about this, if anything? Also, are their situations in which damage to the body is so pronounced that geroprotector use is not likely to substantially change health despite positive changes in an epigenetic clock? Not knowing how epigenetic clocks and geroprotectors impact health and over what time frames calls into question the use of short-term trials of geroprotectors focusing on an epigenetic clock as a primary outcome measure. In fact, the question of how long it might take for a geroprotector to induce health benefits could lead to the almost comical, yet likely true, claim that one could literally die of age-related diseases while waiting for a geroprotector to induce its favorable effects!

## MORE COMPREHENSIVE TRIALS

Given the issues with the use of epigenetic clocks as primary endpoints in clinical trials of geroprotectors described herein, it could be argued that alternative types of studies investigating geroprotectors should be pursued, at least until epigenetic clocks are proven to be reliable surrogate endpoints (for example, in the way that surrogate endpoints for, e.g., cancer and a whole host of other conditions have proven useful [60]). These could include trials of geroprotectors that focus on their ability to impact multiple accepted clinical measures of health (e.g., blood pressure and related hemodynamic measures, immune function assays, muscle function tests, kidney function assays, sleep surveys, mood questionnaires, etc.) in addition to assays interrogating known hallmarks of aging [27]. Currently, regulatory standards for approving an intervention by agencies such as the US Food and Drug Administration (FDA) require an association of an indication or primary endpoint or a singular surrogate for that endpoint with an intervention. Such an association can allow the intervention to be placed into a broader pharmacopeia or formulary for use by clinicians. In this light, clinical trials with multiple endpoints are a rare exception, as discussions surrounding the approval of the TAME trial suggest [24,25,27]. In addition, trials with multiple primary phenotypes can be problematic for statistical reasons (e.g., more opportunities for problems with measurement reliability, greater likelihood of false positive results, etc.).

In addition, trials seeking to vet epigenetic clocks themselves as *bona fide* surrogate endpoints for disease predisposition should be pursued in ways that are analogous to trials exploring the reliability of surrogate endpoints in oncology and other settings [61]. Such trials would not directly benefit tests of a geroprotector, but they could be pursued to directly explore the relationship between multiple accepted clinical health measures, such as lipid levels, or blood pressure, as well as various hallmarks of aging, etc. and epigenetic clock measures. In this light, any interventions used (e.g., exercise, atorvastatin, lisinopril, senolytics, meditation, etc.) in such trials are simply meant to improve specific health measures (e.g., blood pressure or cholesterol level) in order to determine how changes in those health measures affect an epigenetic clock (or vice versa). Other trials could focus on multiple health measures that might be affected by a potential geroprotector. In these trials, the epigenetic clock measures and any other non-vetted biomarkers would be treated as secondary measures to be associated with the clinical measures, with the clinical measures themselves acting as the primary endpoints used to evaluate the effect of the geroprotector [62].

Two concerns with such trials might arise. First, it is arguable that most accepted clinical and subclinical measures are themselves blunt instruments for assessing health and disease risk, thus, many emerging markers derived from various ‘omic’ assays (transcriptomics, epigenomics, proteomics, metabolomics, etc.), imaging protocols, wireless devices, etc. might be better. However, these emerging assays would also have to be assessed for their reliability as surrogate endpoints in relevant clinical trials. Second, as noted, relevant statistical analyses might be complicated for a trial with multiple outcome measures. We do not believe this will be the case, however, as it is well known in the statistical analysis community that if multivariate statistical tests are used to test an omnibus hypothesis that, e.g., all (or most) of the health measures have changed for the better after the administration of a geroprotector, then the study could have *greater* power than a single univariate test focusing on one of those measures if the geroprotector does indeed work as it should [63]. This omnibus test, if the null hypothesis of no effect is rejected, would be consistent with the geroscience hypothesis, since it assumes multiple health outcomes are positively affected by a true geroprotector. There are many designs that could be used in the pursuit of such studies, but aggregated N-of-1 trials are excellent candidates [64–67].

Finally, one could argue that a geroprotector may influence the aging rate, possibly as reflected in an epigenetic clock, in subtle ways that would not manifest in changes in standard blunt-instrument, commonly-used measures of health, such as blood pressure and lipid levels. However, one could ask whether someone should actually trust a geroprotector that supposedly prevents, e.g., stroke or heart disease, but does so with no appreciable effect on blood pressure or cholesterol? Could it be argued, that a particular geroprotector affects some cardiovascular disease-related mechanism that does not bear on blood pressure or lipid levels with no evidence for what this mechanism might be? Could it truly be that a geroprotector, as reflected in its ability to modulate an epigenetic clock and nothing else, renders all signs and symptoms of disease processes benign? Also, which among many measures might one want to consider in relevant multi-endpoint trials of geroprotectors is an open question [62]. However, the intuition that standard proven health measures should be evaluated for geroprotective effects as primary endpoints, with or without an epigenetic clock included in the study, even in short term trials, is a strong one.

## CONCLUSIONS

The geroscience hypothesis is indeed an exciting one, and one that will likely receive considerable attention in the future. Geroprotectors arising from studies exploring the geroscience hypothesis would undoubtedly revolutionize health care and result in dramatic societal changes, and for these reasons should be taken extremely seriously. However, the biomedical science community should be very sensitive to overenthusiasm concerning ways in which geroprotectors are vetted, since reliance on a solitary measure of aging, for example an epigenetic clock, to vet candidate geroprotectors might not be necessary. If geroprotectors, by definition, should improve health during the aging process, and health can be measured in myriad ways, then relevant trials should focus on these health measures directly. In fact, as we have argued, it would be hard to make the case that a geroprotector that is *only* known or shown to modulate an epigenetic clock will extend health span or lifespan without impacting anything associated with health from traditional clinical perspectives. In addition, if one could show that a geroprotector actually does modulate age-related disease processes using routine and accepted clinical measures then the mechanism of action of that geroprotector is likely to be a key to an underlying universal aging clock. Ultimately, a purported geroprotector that has either no observable effect on many available common sense, well-accepted measures of health and vitality, or will only have an effect on health via some cryptic mechanism after the many years of use during which an individual is at typical risk for disease, is a tough sell.

In this light, at least two obvious conclusions and directions should emerge from broader discussions of tests of geroprotectors. First, a new focus on testing a geroprotector’s effects on broadly accepted and even emerging clinically-relevant health measures is appropriate. If successful, that alone should compel the community to take a more serious look at the geroprotector in question, as well as the geroscience hypothesis more broadly, irrespective of accompanying changes in an epigenetic clock. Second, clinical trials such as those envisioned would be an ideal place to vet epigenetic clocks as secondary outcomes. This is the case since relevant trials could be used to assess the potential *causal* relationships between an epigenetic clock and various health measures in earnest. In other words, these relationships would be explored under controlled conditions with longitudinal assessments and relevant hypothesized perturbations in the form of candidate geroprotectors in an appropriately designed and statistically powered setting.
